# Effect of Differences in Month and Location of Measurement in Estimating Prevalence and Trend of Wasting and Stunting in India in 2005–2006 and 2015–2016

**DOI:** 10.1093/cdn/nzaa092

**Published:** 2020-05-23

**Authors:** Emily M Madan, Edward A Frongillo, Sayeed Unisa, Laxmikant Dwivedi, Robert Johnston, Abner Daniel, Praween K Agrawal, Sila Deb, Ajay Khera, Purnima Menon, Phuong H Nguyen

**Affiliations:** 1 Division of Nutritional Sciences, Cornell University, Ithaca, NY, USA; 2 University of South Carolina, Columbia, SC, USA; 3 International Institute for Population Sciences, Mumbai, India; 4 UNICEF, New Delhi, India; 5 Ministry of Health & Family Welfare, New Delhi, India; 6 Poverty and Health Nutrition Division, International Food Policy Research Institute, Washington DC, USA

**Keywords:** stunting, wasting, seasonality, month of year, undernutrition

## Abstract

**Background:**

Child undernutrition in India remains widespread. Data from the National Family Health Survey 3 and 4 (NFHS-3 and NFHS-4) suggest that wasting prevalence has increased while stunting prevalence has declined.

**Objective:**

The objectives of this study were to do the following: *1*) describe wasting and stunting by month of measurement in India in children <5 y of age in NFHS-3 and NFHS-4 surveys, and *2*) test whether differences in the timing of anthropometric data collection and in states between survey years introduced bias in the comparison of estimates of wasting and stunting between NFHS-3 and NFHS-4.

**Methods:**

Data on wasting and stunting for 42,608 and 232,744 children aged >5 y in the NFHS-3 and NFHS-4 survey rounds were analyzed. Differences in the prevalence of wasting and stunting by month of year and by state of residence were examined descriptively. Regression analyses were conducted to test the sensitivity of the estimate of differences in wasting and stunting prevalence across survey years to both state differences and seasonality.

**Results:**

Examination of the patterns of wasting and stunting by month of measurement and by state across survey years reveal marked variability. When both state and month were adjusted, regardless of the method used to account for sample size, there was a small negative difference from 2005–2006 to 2015–2016 in the prevalence of wasting (−0.8 ± 0.6 percentage points; *P* = 0.2) and a negative difference in stunting prevalence (−8.3 ± 0.7 percentage points; *P* < 0.001), indicating a small bias for wasting but not for stunting in unadjusted analyses.

**Conclusions:**

State and seasonal differences may have introduced bias to the estimated difference in prevalence of wasting between the survey years but did not do so for stunting. Future data collection should be designed to maximize consistency in coverage of both time and place.

## Introduction

Physical growth is a useful and widely accepted marker of child health and nutritional status. In India, child undernutrition, assessed as attained growth, is widespread. According to the National Family Health Survey-4 (NFHS-4), the nationwide prevalences of wasting, stunting, and underweight in children <5 y of age in 2015–2016 were 21.0%, 38.4%, and 35.8%, respectively (1). Although the prevalence of stunting has declined since the NFHS-3 survey in 2005–2006 from 48% to 38.4%, the prevalence of wasting appears to have increased from 19.8% to 21% (1, 2). The increase in wasting prevalence was unexpected given continued growth in India's economy, scaling up of nutrition programs during this period, a range of policy and legal actions related to improving the right to food, and no evidence of the worsening of conditions known to be associated with wasting. We asked, therefore, whether there were other, methodological reasons for the observed increase. After considering various potential methodological issues (e.g., data quality, age estimation bias), the most plausible explanation was that the difference in the months of data collection between the 2 surveys may have introduced bias into comparisons of national wasting prevalence. Data for the NFHS-3 and NFHS-4 surveys were collected primarily from December 2005 to August 2006 and from January 2015 to December 2016, respectively (1, 2).

In tropical and subtropical regions of developing countries, monthly and seasonal variation in the growth of children has been well documented. Most evidence suggests a decline in rates of growth that occur during “hungry” periods of the year, which often coincide with the rainy months leading up to the harvest (3–8). This phenomenon in developing countries is thus typically attributed to monthly or seasonal variation in factors such as food availability and infectious disease (9). In Bangladesh, for example, a 3–4-fold difference in the percentage of expected monthly gain in infants 6–60 mo of age was observed in different months of the year (worst during the rainy season and harvest period) (9). In the NFHS-3 survey, child anthropometry was not collected during the late monsoon and harvest months in India (September–November) when peak periods of child wasting are frequently observed in tropical and subtropical regions of developing countries. A period of relatively higher wasting being missed in data collected in NFHS-3 but captured in NFHS-4 possibly explains why wasting prevalence was observed to increase between survey years.

Analyses of NFHS survey data also suggest that substantial variation exists in the estimates of child wasting among states (within each survey) and within states (across surveys). A comparison between the 2 surveys of the timing of data collection and the states where the data were collected reveals varying degrees of overlap in the months of data collection (**Table 1**). For many states, there were some months of overlap of data collection between the survey years. For other states, however, there were no months of overlap (e.g., Madhya Pradesh) (1, 2). The differential timing of data collection overall and within states between the NFHS-3 and NFHS-4 surveys may have affected nationwide estimates of wasting and the observed increase in wasting prevalence over time. Little is known, however, about how subnational variations in data collection temporality affect national prevalence estimates.

**TABLE 1:** Timing of data collection and weighted prevalence of wasting and stunting by state in NFHS-3 and NFHS-4 surveys accounting for household sample weights^1^

	Weighted prevalence of wasting (%)	Weighted prevalence of stunting (%)	2005 or 2015		2006 or 2016											
Surveys by state			Nov	Dec	Jan	Feb	Mar	Apr	May	Jun	Jul	Aug	Sep	Oct	Nov	Dec
India																
NFHS-3	19.8	48.0														
NFHS-4	21.0	38.4														
Andhra Pradesh																
NFHS-3	11.4	39.1														
NFHS-4	16	30.4														
Arunachal Pradesh																
NFHS-3	15.4	41.7														
NFHS-4^2^	18.1	27.6														
Assam																
NFHS-3	14.9	44.4														
NFHS-4	17	36.3														
A & N Islands																
NFHS-3	—	—														
NFHS-4^2^	16.3	27.6														
Bihar																
NFHS-3	27.5	53														
NFHS-4^2^	21.7	48.2														
Chandigarh																
NFHS-3	—	—														
NFHS-4	7.9	40.1														
Chhattisgarh																
NFHS-3	20.2	53.1														
NFHS-4	25	37.9														
NCT New Delhi																
NFHS-3	16	41.1														
NFHS-4	16.8	29.4														
Dadra Nagar Haveli																
NFHS-3	—	—														
NFHS-4	20.4	49														
Daman & Diu																
NFHS-3	—	—														
NFHS-4	39.4	16.2														
Goa																
NFHS-3	15.7	23.5														
NFHS-4^2^	21.1	23.4														
Gujarat																
NFHS-3	20.9	50.6														
NFHS-4	26.9	38.3														
NFHS-3	22.2	46.1														
NFHS-4^2^	23.2	31														
Himachal Pradesh																
NFHS-3	21.7	31.9														
NFHS-4	15.2	26.9														
Jammu and Kashmir																
NFHS-3	14.9	34.1														
NFHS-4	17.9	26.5														
Jharkhand																
NFHS-3	35.7	48.5														
NFHS-4	28.2	44.5														
Karnataka																
NFHS-3	20.3	43.1														
NFHS-4^2^	26.6	34.6														
Kerala																
NFHS-3	16.2	19.6														
NFHS-4	14.9	20														
Lakshadweep																
NFHS-3	—	—														
NFHS-4	19.6	36.7														
Madhya Pradesh																
NFHS-3	42.4	45.4														
NFHS-4^2^	25.7	41.4														
Maharashtra																
NFHS-3	18.6	43.4														
NFHS-4^2^	25.5	35.8														
Manipur																
NFHS-3	13	36.3														
NFHS-4^2^	6.4	31.7														
Meghalaya																
NFHS-3	32.3	44.8														
NFHS-4^2^	16.3	43														
Mizoram																
NFHS-3	7.3	42														
NFHS-4	8.3	30														
Nagaland																
NFHS-3	15.7	39.4														
NFHS-4	15.2	27.7														
Odisha																
NFHS-3	19.9	41.9														
NFHS-4	23.4	37.8														
Pondicherry																
NFHS-3	—	—														
NFHS-4^2^	32.2	38.7														
Punjab																
NFHS-3	11	35.7														
NFHS-4	15.3	22.8														
Rajasthan																
NFHS-3	25.8	42														
NFHS-4	23.2	38.3														
Sikkim																
NFHS-3	10.9	41.1														
NFHS-4^2^	15.9	30.5														
Tamil Nadu																
NFHS-3	25.9	36.9														
NFHS-4^2^	20.9	25														
Tripura																
NFHS-3	28.8	33.3														
NFHS-4^2^	17.9	23.4														
Uttar Pradesh																
NFHS-3	16.9	56.6														
NFHS-4^2^	20.2	45.7														
Uttarakhand																
NFHS-3	16.9	43.1														
NFHS-4^2^	22.9	32.4														
West Bengal																
NFHS-3	14.4	41														
NFHS-4	19.3	33.2														

1 NFHS, National Family Health Survey.

2 Months in 2015–2016.

Note: Blue bars represents data collected in 2006; green bars represents data collected in 2016.

The objectives of this study were therefore to describe wasting and stunting by month of measurement in India in children <5 y of age in the NFHS-3 and NFHS-4 surveys, and to estimate the magnitude of any bias introduced in the comparison of estimates of wasting and stunting between the NFHS-3 and NFHS-4 surveys by differences between survey years in the timing of anthropometric data collection and in the states where data were collected.

## Methods

### Sampling and study populations

We used data on 42,608 and 232,744 children aged <5 y in the NFHS-3 and NFHS-4 survey rounds, respectively. Both NFHS-3 and NFHS-4 provide nationally representative estimates by surveying the 29 states in India that comprise 99% of the country's population. NFHS-4 was designed to provide district-level estimates, while NFHS-3 was designed to provide only state-level estimates (1, 2). Both rounds of NFHS data provide information on date of birth, height, weight, and date of measurement. The detailed methodology for conducting NFHS surveys is described elsewhere (1, 2). We excluded from the analysis any children with extreme or missing values for weight-for-height *z* score (WHZ; <−5 or >5), height-for-age *z* score (HAZ; <−6 or >6), and weight-for-age *z* score (<−6 or >5), as recommended by WHO (10). Some individuals may have met >1 exclusion criterion.

### Child growth measures

Information about the instruments and protocols for anthropometric measurements used in NFHS-3 and NFHS-4 is available elsewhere (1, 2). In brief, trained enumerators, working in pairs, collected the anthropometric measures in duplicate. Weight was measured with portable seca electronic scales. Recumbent length was measured in children <24 mo old with Shorr boards in NFHS-3 and seca infantometers in NFHS-4, and seca stadiometers were used to measure standing height in children ≥ 24 mo old. Child wasting was defined as WHZ <−2 SD and child stunting was defined as HAZ <−2 SD.

### Statistical analysis

The weighted prevalence of wasting and stunting was computed for the total samples of children <5 y of age in NFHS-3 and NFHS-4 data and by month and year. Scatter plots were produced to describe patterns of wasting and stunting across months of measurement between survey years. Weighted prevalence of wasting and stunting was calculated by state and by year to examine changes within states over time. These data were used in the primary analyses which compared the magnitude and *P* value of the coefficient for the year variable (i.e., 2005–2006 compared with 2015–2016) estimated from a set of multiple regression models that either excluded or included state and month of measurement. Specifically, to examine whether estimation of the difference in prevalence from 2005–2006 to 2015–2016 was affected by month of measurement, regression analyses were conducted to test the sensitivity of the estimate of the difference in wasting and stunting prevalence across survey years to both state differences and seasonality (i.e., months of the year when the measurement was taken). In these regression models, the dependent variable was the prevalence of wasting or the prevalence of stunting for children. The observations in the data set were wasting and stunting prevalence by year, state, and month, which were calculated accounting for household sampling weights. Year (2005–2006 or 2015–2016), state, and month of year were considered as nominal, fixed-effect, independent variables. These regression models produced theoretically unbiased estimates of coefficients despite the empty cells in the data matrix defined by state and year under the assumption of no interaction between state and month (11) (Table 1). The unadjusted coefficient for year was compared to coefficients from models adjusted for only state, only month, or both state and month. Models were run initially without accounting for variations in the size of the samples for the surveys for each year, state, and month. Two sets of additional models were then run, each set using a different method to account for variation in sample size. One method included the square root of the sample size as a covariate and the other used the sample size as an analytic weight.

To further test the robustness of these models, all of these analyses were replicated on datasets that were restricted to 1) months when data were collected in both survey years (December–August) and 2) states where data were collected in both survey years (6 states excluded). These analyses necessarily assumed that that there were no interactions among year, state, and month since the data did not allow tests of potential interactions.

Analyses conducted at the aggregate level for the state were replicated at the individual level using procedures for complex samples that incorporated the primary sampling units and household weights. Household weights were normalized within each year before pooling the weights. In addition to models that accounted for year, year and state, year and month, and all 3, a model was run further adjusting for child's age, sex, state, caste of the mother, religion of mother, any morbidity, having no toilet, education of mother, place of residence, and mother's age. These variables were chosen because they are known risk factors for poor child growth outcomes (12, 13). Data were analyzed using Stata version 13 (Stata Corporation, 2005).

## Results

**FIGURE 1:**
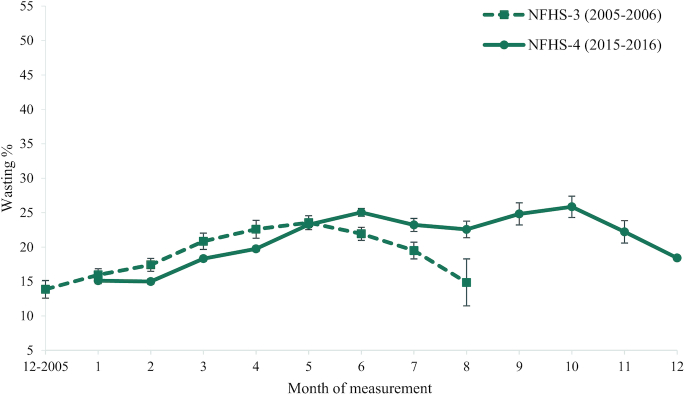
Weighted prevalence of wasting in India in NFHS-3 and NFHS- 4 India by month of measurement. Data presented as mean estimate and 95% CI. NFHS, National Family Health Survey.

The weighted prevalence of wasting in India was lower (15–17%) between December and February in both survey years and then increased (18–23%) between March and May in both survey years (**Figure 1**). In data from NFHS-3, the weighted prevalence of wasting peaked in May (23%), while in NFHS-4, the prevalence of wasting peaked in June (25%), and then again in September and October (26%), months of the year when data were not collected in NFHS-3.

An examination of the prevalence of wasting within states across survey years showed marked variability (Table 1). In some states, increases in wasting prevalence were observed between survey years (e.g., in Goa, the prevalence of wasting increased from 15.7 to 21.1%), while in other states, decreases were observed (e.g., in Himachal Pradesh wasting prevalence decreased from 21.7% to 15.2%).

When both state and month were adjusted, regardless of the method used to account for sample size, there was a small negative difference from 2005–2006 to 2015–2016 in the prevalence of wasting, i.e., the prevalence decreased from 2005–2006 to 2015–2016 by 0.8 percentage points (−0.8 ± .6; *P* = 0.2) (**Table 2**). In contrast, the unadjusted model with only year as an independent variable had a positive coefficient, suggesting the prevalence of wasting increased by 1.7 percentage points (1.7 ± 1.0; *P* = 0.082). The *P* values for year in each of the 4 models were ≥0.08. Examination of the root mean square errors and percentage of variance explained for each model showed that year alone explained little of the variance in wasting prevalence, month explained a modest amount of the variance, and state explained a substantial amount of the variance. The results from the 2 robustness analyses that restricted the sample were essentially the same as those reported here (not shown). Analyses replicated at the individual level showed a pattern similar to that observed in analyses at the aggregate level, but with lower *P* values (Table 2).

**TABLE 2:** Year coefficients, standard errors, and *P* values for various regression models of weighted wasting prevalence (in percentage points) as the dependent variable at the aggregated level and at the individual level, accounting for household sample weights

Models data included	Aggregated analyses with sample size as analytic weight (*n *= 343)	*P* value	Individual-level analyses (*n *= 275,352)^1^	*P* value
Year	1.7 ± 1.0	0.08	0.9 ± 0.4	0.02
Year, state	0.6 ± .0.7	0.4	0.9 ± 0.4	0.01
Year, month	0.6 ± .010	0.6	−2.1 ± 0.4	<0.001
Year, state, month	−0.8 ± 0.6	0.2	−1.8 ± 0.4	<0.001
Year, state, month^1^	—	—	−0.6 ± 0.4	0.14

1 Adjusted for child's age, sex, state, caste of mother, religion of mother, toilet, education of mother, place of residence, and mother's age.

**FIGURE 2:**
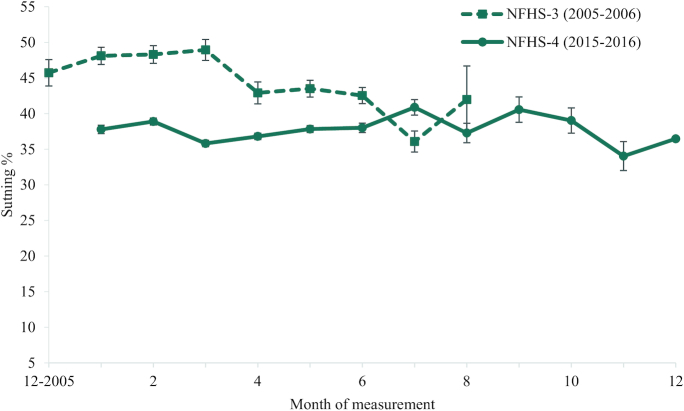
Weighted prevalence of stunting in India in NFHS-3 and NFHS-4 India by month of measurement. Data presented as mean estimate and 95% CI. NFHS, National Family Health Survey.

The weighted prevalence of stunting in India in NFHS-3 decreased from March to July (from 48% to 36%). In the NFHS-4 survey, the weighted prevalence of stunting remained relatively stable throughout the year (about 37% stunted) (**Figure 2**).

In regression analyses to test the sensitivity of the estimate of the difference in stunting prevalence between survey years to differences in stunting prevalence by month of year and by state, regardless of the method used to account for sample size, there was a negative difference in stunting prevalence from 2005–2006 to 2015–2016 of 6–8 percentage points (−8.3 ± 0.7; *P* < 0.001 in the model adjusting for year, month, and state). (**Table 3**). Analyses replicated at the individual level show a pattern like that in analyses at the aggregate level.

**TABLE 3:** Year coefficients, SE, and *P* values for various regression models of weighted stunting prevalence (in percentage points) as the dependent variable at the aggregated level and at the individual level, accounting for household sample weights

Models data included	Aggregated analyses with sample size as an analytic weight (*n *= 343)	*P* value	Individual-level analyses (*n *= 275,071)^1^	*P* value
Year	−6.4 ± 1.2	<0.001	−9.6 ± 0.5	<0.001
Year, state	−8.8 ± 0.6	<0.001	−9.4 ± 0.5	<0.001
Year, month	−5.8 ± 1.3	<0.001	−9.0 ± 0.6	<0.001
Year, state, month	−8.3 ± .007	<0.001	−8.5 ± 0.5	<0.001
Year, state, month^1^	—	—	−5.4 ± 0.5	<0.001

1 Adjusted for child's age, sex of the child, state, caste of the mother, religion of mother, toilet, education of mother, place of residence, mother's age.

## Discussion

We examined patterns of wasting and stunting by month of year in NFHS data from India to assess the possible bias introduced into analyses that compare wasting and stunting prevalence across survey years without accounting for month of year. These analyses suggest that the differential timing of data collection across states between 2005–2006 and 2015–2016 NFHS surveys likely introduced a bias to analyses that compare the prevalence of wasting between survey years but not in analyses that compare the prevalence of stunting.

The variance explained by month of year in analyses of wasting prevalence was greater than the amount of variance explained by year alone but smaller than the amount of variance explained by state. Several studies in India and elsewhere have documented differences in the prevalence of wasting by month of year in children <5 y of age. The patterns of stunting by month of year, however, are less consistent (6, 9, 14), likely because cumulative indicators such as stunting are poor measures of short-term changes in linear growth compared to, for example, linear growth velocity (13). In NFHS data, the prevalence of stunting was observed to be less variable throughout the year than the prevalence of wasting. Therefore, the potential for the month of measurement to bias comparisons of stunting prevalence between survey years may have been less.

These analyses reveal that, even after adjustments for month of measurement and state, the findings for the prevalence of wasting in India in 2015–2016 remain high; the weighted wasting prevalence in 2015–2016 ranged from 17% to 49% across states (Table 1). Based on these analyses, although the prevalence of wasting may not have increased in India, it has also not decreased at nearly the same rate as stunting. Several recent analyses have explored the possible interrelationships between wasting and stunting and have highlighted many of the overlapping determinants of types of growth faltering (13, 15). Continued efforts are needed to address major risk factors for undernutrition throughout the year, such as low maternal BMI, anemia, and micronutrient deficiencies, which remain high in India (16, 17).

One strength of our analyses is that we did not make any assumptions about pattern of prevalence across months of the year. That is, no continuous function was assumed for the relationship between months and prevalence. These results, then, directly derive from the data available for each survey year, state, and month, avoiding any potential misspecification of a continuous function. These analyses suggest that when both the differences among states and between months of the year are adjusted, wasting prevalence decreased slightly from 2005–2006 to 2015–2016, in contrast to previously reported findings (1, 2). Our results were intended to test the sensitivity to accounting for state and month, however, and were not intended to produce nationally representative estimates of the wasting prevalence or difference in prevalence across the 2-y timespan of the study. To do so would require the development of further analysis methods that incorporate sampling weights and population sizes, which may not be possible given that the limitations in the availability of data across states and months prevent comparisons between the 2 surveys.

In conclusion, the observed increase in wasting prevalence between NFHS-3 and NFHS-4 surveys in India (1, 2) may be partially explained by differences in the prevalence of wasting by both state and month of year. If data had been collected in the same period of the year in every state across India, a small reduction of wasting, rather than an increase, may have been observed. The difference between estimates of 2.5 percentage points in wasting is >10% of the national prevalence estimate for wasting, large enough to be of public health importance. The difference in direction is of both public health and political importance given that trends in nutrition program coverage and other measures of child nutrition improved over time (1).

The narrative of progress related to nutrition in India has been built around the reduction of stunting and underweight, but our findings suggest that it is not clear that wasting has worsened, as noted from the use of the raw prevalence estimates. This problem is not restricted to India; it arises from the challenges of doing national surveys in countries, even high-income countries (e.g., the US NHANES), where data can only be collected in some locations during some seasons. The 2019 WHO/UNICEF report on recommendations for the data collection of anthropometric indicators in children <5 y of age suggests that during survey planning, researchers “identify the best period to implement the survey to allow comparison with previous surveys,” but provides little guidance for the practical implications of this suggestion (18). The best strategy to mitigate temporal instability of estimates in the future is to plan data collection to have the maximum possible coverage and consistency of coverage in terms of time (i.e., months of year) and place (e.g., states). The feasibility and cost of doing so must be carefully considered relative to the magnitude of possible biases introduced into comparisons of anthropometric data between surveys that are not consistent over time in data collection by location and month.

## Notes

Supported by funding from the Bill & Melinda Gates Foundation, through POSHAN, led by the International Food Policy Research Institute.

Author disclosures: The authors report no conflicts of interest.

Abbreviations used: HAZ, height-for-age *z*-score; NFHS, National Family Health Survey; WHZ, weight-for-height *z*-score.
